# Novel SUMO-Protease SENP7S Regulates β-catenin Signaling and Mammary Epithelial Cell Transformation

**DOI:** 10.1038/srep46477

**Published:** 2017-04-21

**Authors:** Samaneh Karami, Feng-Ming Lin, Santosh Kumar, Shaymaa Bahnassy, Hariprasad Thangavel, Maram Quttina, Yue Li, Jing Ren, Tasneem Bawa-Khalfe

**Affiliations:** 1 Center for Nuclear Receptors and Cell Signaling, Department of Biology & Biochemistry, University of Houston, Houston, Texas, 77204, USA; 2 Department of Cardiology, University of Texas MD Anderson Cancer Center, Houston, Texas, 77030, USA; 3 Texas Heart Institute at St. Luke’s Episcopal Hospital, Houston Texas, 77030, USA; 4 Department of Integrative Biology and Pharmacology, The University of Texas Health Science Center at Houston, Houston, TX, 77030, USA

## Abstract

SUMO post-translational modification of proteins or SUMOylation ensures normal cell function. Disruption of SUMO dynamics prompts various pathophysiological conditions, including cancer. The burden of deSUMOylating the large SUMO-proteome rests on 6 full-length mammalian SUMO-proteases or SENP. While multiple SENP isoforms exist, the function of these isoforms remains undefined. We now delineate the biological role of a novel SENP7 isoform SENP7S in mammary epithelial cells. SENP7S is the predominant SENP transcript in human mammary epithelia but is significantly reduced in precancerous ductal carcinoma *in situ* and all breast cancer subtypes. Like other SENP family members, SENP7S has SUMO isopeptidase activity but unlike full-length SENP7L, SENP7S is localized in the cytosol. *In vivo,* SUMOylated β-catenin and Axin1 are both SENP7S-substrates. With knockdown of SENP7S in mammary epithelial cells, Axin1-β-catenin interaction is lost and β-catenin escapes ubiquitylation-dependent proteasomal degradation. SUMOylated β-catenin accumulates at the chromatin and activates multiple oncogenes. Hence, non-tumorigenic MCF10-2A cells with reduced SENP7S exhibit greater cell proliferation and anchorage-dependent growth. SENP7S depletion directly potentiates tumorigenic properties of MCF10-2A cells with induction of anchorage-independent growth and self-renewal in 3D-spheroid conditions. Collectively, the results identify SENP7S as a novel mediator of β-catenin signaling and normal mammary epithelial cell physiology.

Posttranslational modifications (PTM) ensure proteomic diversity within a cell. Many proteins that modulate normal cell function are targets for SUMO-PTM or SUMOylation. SUMO-specific proteases (SENP) readily reverse SUMOylation to maintain equilibrium of SUMOylated/unmodified proteins within a cell^1^. Maintaining SUMO dynamics is critical as SUMO-PTM of a substrate directs protein activity, interaction with other molecules, subcellular localization, and/or stability^2^. With novel proteomic approaches, the number of identified cellular targets of SUMOylation is increasing expeditiously^3^. While canonical studies primarily confined SUMO-PTM to nuclear proteins, more current reports demonstrate SUMOylation of multiple non-nuclear proteins. In contrast, the SENP family resides predominantly in the nucleus. Hence, it is unclear what modulates the SUMOylation status of proteins outside the nucleus.

Recently we identified a shorter splice variant of SENP7, SENP7S (NM_001077203.2) that is transcribed in human mammary epithelia^4^. As compared to the full-length transcript SENP7L, SENP7S includes the catalytic domain but lacks exon 6. Alternative splicing events maintain an inverse proportion of SENP7S to SENP7L as observed in breast cancer (BCa) patient samples. Gain of SENP7L correlates with onset of metastatic disease and directs epigenetic remodeling for epithelial-mesenchymal transition in BCa cells^4^. Although a concurrent loss of SENP7S is reported, the biological function of SENP7S remains undefined. A genome-wide siRNA screen suggests targeted knockdown of the SENP7 gene transcript NM_001077203.2 alters Wnt-activated β-catenin signaling in a sarcoma cell line^5^. How this SENP7S variant regulates β-catenin signaling was not reported. Specifically, it is unclear whether SENP7S deSUMOylates β-catenin and/or other mediators of the β-catenin cascade to initiate this change in β-catenin signaling.

In mammary epithelial cells, β-catenin is produced excessively to maintain cell-cell adhesion at the membrane and initiate gene transcription upon nuclear translocation. Nuclear β-catenin accumulation occurs with activation of the canonical Wnt pathway and contributes to aberrant proliferation. Constitutive nuclear translocation of β-catenin in the mouse mammary gland potentiates the self-renewal property of luminal mammary epithelial cells and BCa development^6^,^7^. Consistently, enhanced cytoplasmic and nuclear β-catenin staining is readily observed in ductal carcinoma and precursor ductal carcinoma *in situ* (DCIS^8^,^9^,^10^). Hence, to maintain physiologically relevant levels of β-catenin, the scaffold protein Axin binds β-catenin, which initiates GSK3β-dependent phosphorylation, subsequent ubiquitylation, and proteasomal degradation.

SUMO-PTM is known to impact β-catenin transcriptional activity^5^,^11^. In fact, β-Catenin and members of the β-catenin destruction complex, Axin1 and GSK3β, are targets for SUMO-PTM^12^,^13^. A recent report suggests SUMOylated β-catenin is resilient to ubiquitin-mediated protein degradation^14^. However, it is unknown if and how β-catenin SUMOylation disrupts association with components of the destruction complex. Additionally, SUMO deconjugation/conjugation factors that dictate the dynamics of β-catenin SUMOylation remain undefined.

In the present manuscript, we demonstrate that SENP7S is a functional SUMO isopeptidase that deSUMOylates β-catenin and Axin1. The loss of SENP7S perturbs translocation of Axin1 to the nucleus, Axin1-β-catenin interaction, and consistently ubiquitylation of β-catenin. SENP7S directs transcription of β-catenin-responsive genes, anchorage-dependent and -independent proliferation, and self-renewal properties of mammary epithelial cells. Collectively, the data defines a biological role for the SENP7S variant in the maintenance of normal mammary epithelial cell physiology.

## Results

### SENP7S is highly expressed in normal mammary epithelia

**Figure 1:**
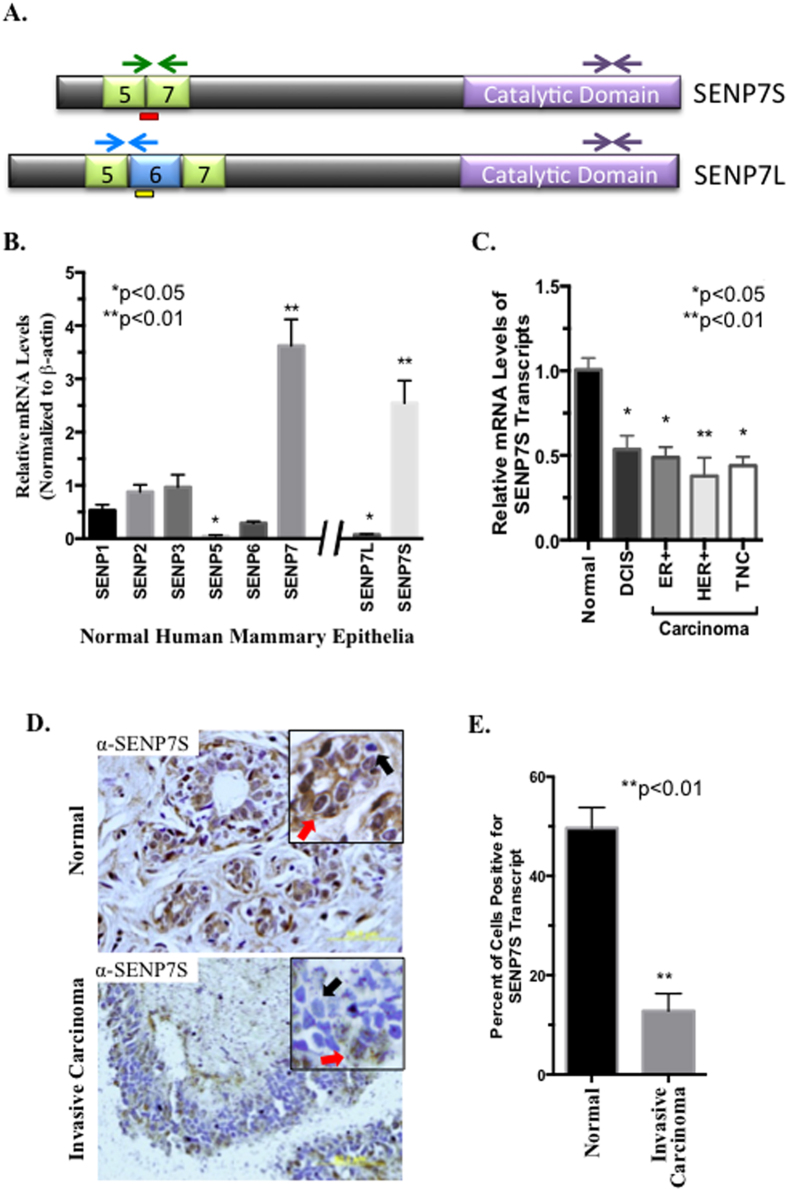
Inverse correlation of SENP7S and SENP7L isoforms in NME, DCIS, and multiple BCa subtypes. (**A**) Schematic illustrating SENP7S and SENP7L isoforms. Green arrows highlight primers for SENP7S, SENP7L-specific primers are indicated with blue arrows, and purple arrows represent primers that detect both isoforms. (**B**,**C**) Real-time PCR analysis of human patient samples in the TissueScan Breast Cancer Survey (Origene) was used to compare all SENPs in NME samples (n = 5, (**B**) or SENP7L and SENP7S in the indicated BCa subtype (n = 35 (**C**)). (**B**) Relative mRNA levels are normalized to the housekeeping gene β-actin and values on the y-axis represent 2^−ΔCt^ times 10^4^. (**C**) Relative mRNA levels are 2^−ΔΔCt^ values and represents expression at a specific BCa type versus normal patient samples. ANOVA and Tukey’s post-hoc analysis and p-values (GraphPad) were based on raw ΔCt, and not 2^ΔΔCt^ values. (**D**) SENP7S assessed in normal and carcinoma human tissue. IHC with SENP7S-specific antibody and hematoxylin counterstain was performed on human tissue samples (normal, n = 18 and carcinoma, n = 19). SENP7S-positive (red arrows) versus -negative (black arrows) cells are highlighted. (**E**) The graph represents the mean±SEM of the percent of SENP7S-positive to total cells in 40X images acquired from normal (n = 6) and carcinoma (n = 7).

Using Taqman primers for exon 20–21 in the catalytic domain of SENP7 (purple arrows, Fig. 1A and Table S1), we observe that in normal mammary epithelia (NME) SENP7 is more efficiently transcribed than the other 5 SENPs (n = 5, Fig. 1B). Further assessment with isoform specific primers reveals short exon-6-deficient SENP7S isoform (NM_001077203.1; green arrows, Fig. 1A) constitutes the majority of SENP7 population. In contrast, the exon-6-expressing full-length SENP7L (NM_020654.3; blue arrows, Fig. 1A) is amongst the least transcribed deSUMOylase in NME (n = 5, Fig. 1B).

Samples from patients with pre-cancerous DCIS lesions exhibit significantly lower levels of the SENP7S message as compared to normal patient samples (n = 9 and 5, respectively, *p* < *0.05*, Fig. 1C). Loss of SENP7S transcripts persists in multiple BCa subtypes (ER+, n = 23; Her2+, n = 7; triple-negative cancer TNC, n = 4, *p* < *0.05*, Fig. 1C); SENP7S mRNA is reduced approximately 2-fold or greater in all BCa samples (*p < 0.05, Fig. 1C). Similarly, the non-cancerous mammary epithelial cell line MCF10-2A expresses greater SENP7S mRNA than ER+ MCF7 and TNC MDA-MB-231 BCa cells (Supplementary Fig. S1).

Subsequently, isoform-specific antibodies were generated for SENP7S or SENP7L epitopes as described in Materials and Methods (red or yellow bar under the respective SENP7 isoform Fig. 1A) and validated via Western Blot experiments (Supplementary Fig. S2A). Evaluation of normal human mammary samples indicates SENP7S protein is readily observed in mammary luminal epithelial cells (red arrow, Fig. 1D). In contrast, SENP7S is significantly reduced in the multi-layer epithelia of invasive carcinoma samples (**p < 0.01, t-test, Fig. 1D,E), corresponding with Fig. 1B mRNA studies. Consistently, higher SENP7S protein is observed in established MCF10-2A than MCF7 cells (Supplementary Fig. S2B). Interestingly an inverse expression for SENP7L is observed in human patient samples and established BCa cell lines; specifically SENP7L mRNA and protein is low in normal epithelia and high in BCa (Supplementary Fig. S2C,D). Collectively the results highlight the loss of SENP7S, but not SENP7L, with the onset of BCa.

### SUMO2/3-specific Isopeptidase Activity of SENP7S

**Figure 2:**
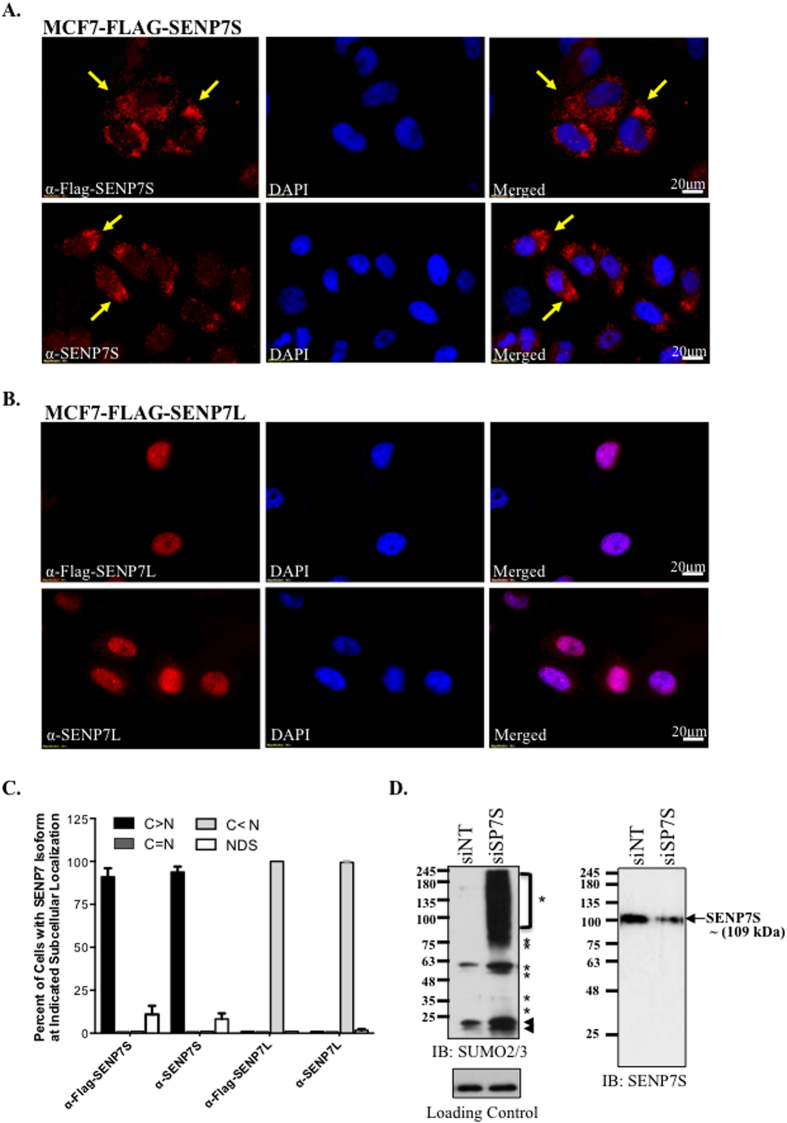
SENP7S is an active SUMO Protease that is located in the cytosol. (**A–C**) MCF7 cells were transfected with either Flag-tagged SENP7S (**A**) or Flag-tagged SENP7L (**B**). Immunofluorescence studies were performed with either Flag or SENP7 isoform-specific antibodies; DAPI was used to identify the nucleus. Yellow arrows highlight the cytosol SENP7S. (**C**) Cells from immunofluorescence images (n = 6 per group) were counted and the subcellular distribution of the SENP7 isoform was evaluated as more cytosolic (C > N), more nuclear (C < N), or equal distribution (C = N). If the image presented a DAPI stain but no antibody fluorescence signal, then the cell was designated as “no detectable signal” (NDS). The graph represents the percent of cells with the indicated subcellular distribution of protein identified with an antibody for Flag (α-Flag-SENP7S or α-Flag-SENP7L), SENP7S (α-SENP7S), or SENP7L (α-SENP7L). (**D**) siRNA was designed for targeted-knockdown of SENP7S (siSP7S). MCF10-2A cells were treated with siSP7S or siNT control for 48hr and whole-cell lysates (WCL) were harvested and immunoblotted (IB) with the indicated antibodies. Global SUMO2/3-conjugated proteins are highlighted with asterisks. Black arrowheads mark the unmodified SUMO2/3.

Assessment of human patient samples and transfected MCF7 cells suggested alternative subcellular localizations of the two isoforms (Fig. 1C and Supplementary Fig. S2C,D). Endogenous SENP7S is heavily localized in the cytosol in both normal and carcinoma samples (red arrow, Fig. 1D). Consistently ectopic Flag-tagged SENP7S in MCF7 cells distributes outside the nucleus as evident in immunofluorescence studies with either the Flag or SENP7S antibody (yellow arrows, Fig. 2A). In contrast, SENP7L appears almost exclusively in the nucleus of mammary epithelia in patients with invasive carcinoma (red arrow, Supplementary Fig. S2C,D) and MCF7 cells (Fig. 2B); this is consistent with its ability to bind multiple chromatin-bound proteins^4^,^15^,^16^,^17^. Assessment of additional images confirmed the inverse subcellular localization of SENP7S versus SENP7L; SENP7S is predominantly cytosolic and SENP7L is nuclear (Fig. 2C). Fractionation studies also indicated similar distribution of the SENP7 isoforms in MCF7 and MCF10-2A cells (Supplementary Fig. S3).

Although the SENP7S shares the same catalytic domain with SENP7L, it is unknown if this short isoform exhibits SUMO isopeptidase activity. To test endogenous isopeptidase activity of SENP7S, we introduced targeted siRNA that efficiently lowered SENP7S in MCF10-2A cells (Fig. 2D) but not SENP7L or other nucleoplasm SENP (Supplementary Fig. S4). Reduction of SENP7S increases global SUMO2/3 conjugation (Fig. 2D) in non-cancerous mammary epithelial cells. Hence SENP7S is required to maintain multiple cellular proteins in the unmodified or deSUMOylated state.

### SENP7S deSUMOylates β-catenin and Axin

**Figure 3:**
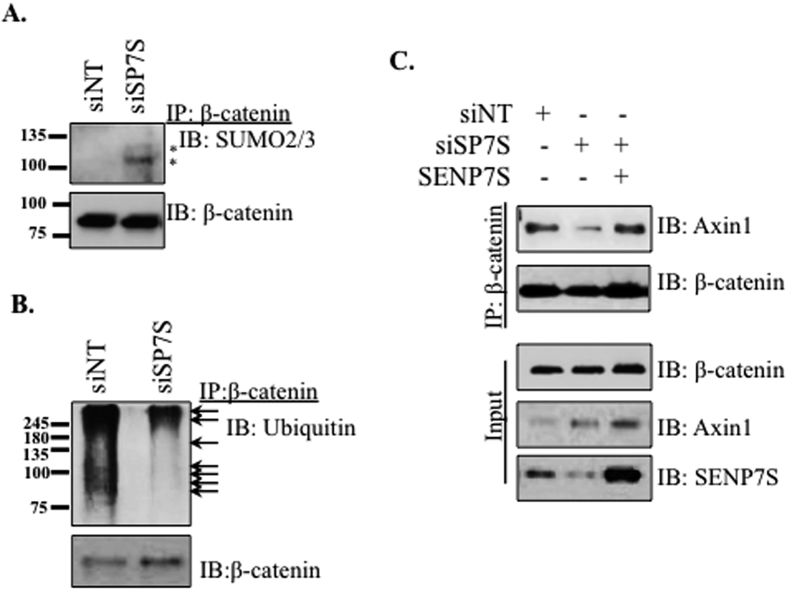
SENP7S regulates SUMOylation and Ubiquitylation of β-catenin. (**A**,**B**) Endogenous β-catenin was immunoprecipitated from MCF10-2A cells that were treated with the indicated siRNA for 48hr. (**A**) SUMO2/3-modified β-catenin could be detected with the SUMO2/3 antibody at the anticipated molecular weight; asterisks are placed adjacent to SUMOylated β-catenin. (**B**) Immunoblots of the immunoprecipitated β-catenin with an anti-ubiquitin antibody revealed loss of the poly-ubiquitin chains with SENP7S-targeted knockdown as highlighted with black arrows. (**C**) Exogenous SENP7S was transfected concurrently to supplement for siRNA-targeted loss of endogenous SENP7S in MCF10-2A cells. β-catenin was immunoprecipitated and interaction with Axin1 was assessed following SDS-PAGE and immunoblotting with an Axin1-specific antibody.

Since β-catenin is a target for SUMOylation and possibly SENP7S^5^,^14^, we next evaluated whether SENP7S loss affects β-catenin SUMO-PTM in MCF10-2A cells. Pull-down of endogenous β-catenin indicated a modest induction of SUMOylated β-catenin in SENP7S- versus non-targeting-siRNA treated cells (Fig. 3A) identifying β-catenin as a target for SENP7S’s isopeptidase activity. Interestingly, the knockdown of SENP7S also reduces the ubiquitylation of β-catenin (Fig. 3B). In the MCF7 BCa cell line, β-catenin ubiquitylation is also minimized following treatment with an alternative siRNA that initiates SENP7S loss (Supplementary Fig. S5A). Hence, the SUMO protease SENP7S can dictate ubiquitin-PTM of β-catenin in non-cancerous and cancerous mammary epithelial cells.

Association with Axin is the initial step for β-catenin ubiquitylation and therefore, β-catenin-Axin1 interaction was determined with SENP7S reduction. Loss of SENP7S decreases interaction between β-catenin and the scaffolding protein Axin1 in MCF10-2A (Fig. 3C) and MCF7 cells (Supplementary Fig. S5B). Rescue with exogenous SENP7S re-establishes association between the two proteins (Fig. 3C) suggesting that SENP7S directs β-catenin-Axin1 binding and consistently β-catenin ubiquitylation.

**Figure 4:**
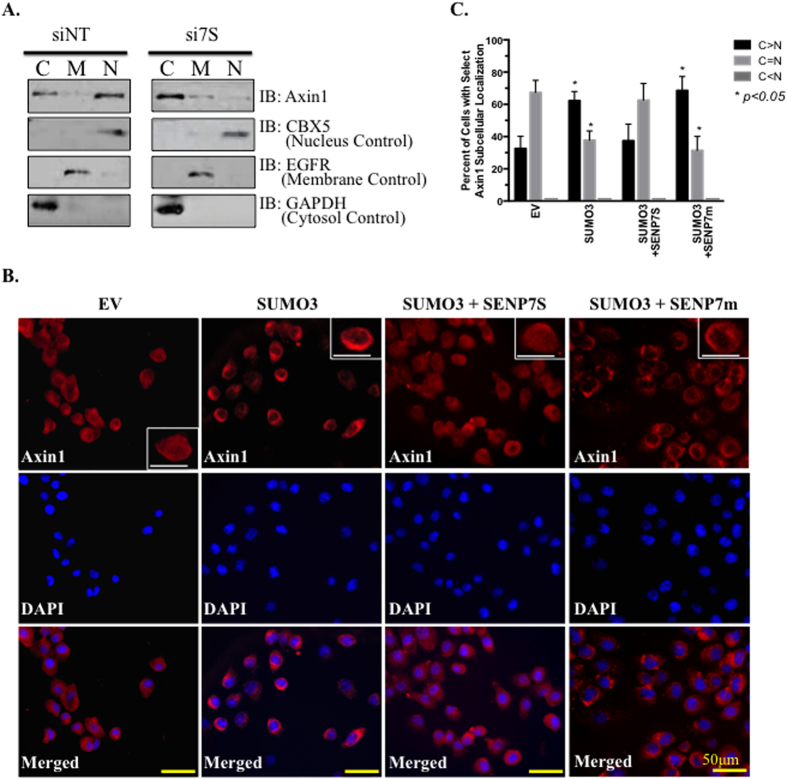
SENP7S dictates the nuclear localization of Axin. (**A**) To identify Axin1, proteins from cytoplasmic (C), cell membrane (M), and nuclear (N) fractions from MCF10-2A cells treated with siSENP7S and siNT (control) were subject to western blot analysis. CBX5, EGFR, and GAPDH served as a loading control for the nuclear, membrane, and cytoplasmic fraction, respectively. (**B**) MCF10-2A cells were seeded on to coverslips and transiently transfected with either empty vector (EV) or SUMO3, or co-transfected with SUMO3 in the presence of wild-type SENP7S or mutant SENP7 (SENP7m). After 24 hrs, cells were stained with an Axin1 antibody (red) and DAPI (blue). Images were acquired by fluorescence microscopy using a 20X objective. Insets represent enlarged cells. (**C**) Cells from immunofluorescence images (**B**) were counted and categorized based on their Axin1 cytosolic (C) and nuclear (N) localization and the number of cells were presented as percentages. C < N staining was not detected in any group. Data shown as mean ± SEM of two independent experiments. One-way ANOVA was used to compare between groups and p < 0.05 was considered significant.

Like β-catenin, Axin1 is subject to SUMO-PTM^13^ and loss of SENP7 is sufficient to potentiate Axin1 SUMOylation (Supplementary Fig. S5C). Interestingly, the same conditions do not initiate GSK3β SUMOylation in MCF10-2A cells suggesting that GSK3β is not a substrate for deSUMOylating activity of SENP7S (data not shown). HyperSUMO conditions achieved with knockdown of SENP7S alters the subcellular localization of Axin1. Specifically, siRNA-targeted SENP7S loss enriches Axin1 in the cytosol while depleting nuclear Axin1 pools (Fig. 4A). Concurrently, modest induction of Axin1 in membrane fraction also occurs with downregulation of the short SENP7 isoform (Fig. 4A). Analogous to fractionation studies, Axin1 is found in the cytosol and nucleus of MCF10-2A cells under normal/control conditions via immunofluorescence (Fig. 4B,C). Cells with this diffused Axin1 distribution are lost with overexpression of SUMO3 (Fig. 4B). Instead, the proportion of Axin1 in the cytosol is significantly increased under these hyperSUMO conditions (*p* < *0.*05, Fig. 4B,C). Concomitant induction of SENP7S, but not the catalytic inactive SENP7 mutant, reduces the number of cells with predominantly cytosolic Axin1 (*p* < *0.*05, Fig. 4B,C). Protein expression under the described conditions was validated via Western Blot (Supplementary Fig. S5D).

### SENP7S regulates β-catenin signaling and mammary epithelial cell transformation

**Figure 5:**
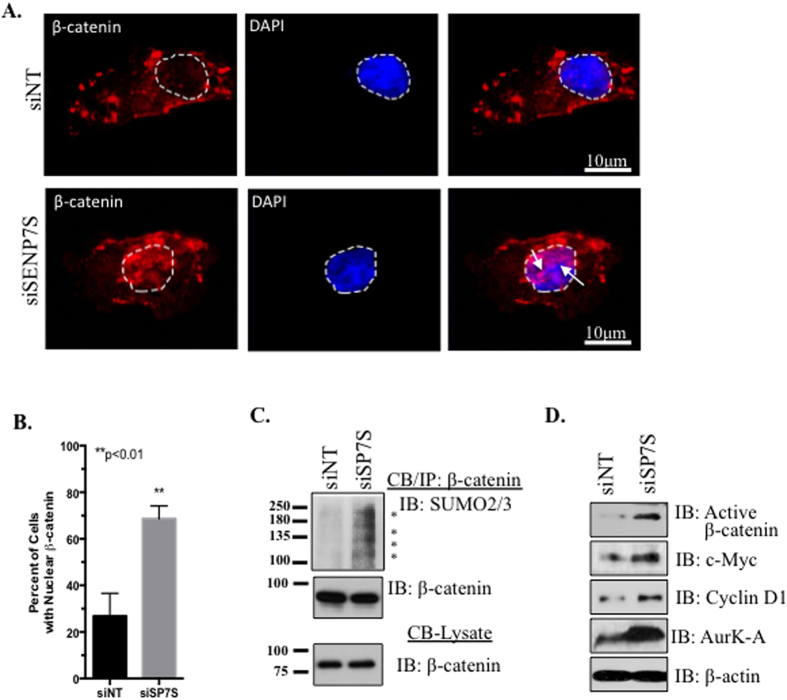
Increased chromatin recruitment of SUMOylated β-catenin and induction of β-catenin-regulated genes. (**A**,**B**) Immunofluorescence was utilized to detect subcellular localization of β-catenin in MCF102-A cells following 48hrs with the indicated siRNA treatment. β-catenin distribution (red) was compared against the nuclear marker DAPI (blue); the white dashed line highlights the DAPI-stained nucleus. White arrows illustrate nuclear enrichment of β-catenin, which is significantly elevated in SENP7S-siRNA treated cells. Data shown as mean ± SEM of three independent experiments. Student’s t-test was used to compare between groups and p < 0.01 was considered significant. (**C**) Chromatin-bound (CB) β-catenin was isolated from treated cells and subject to immunoblot for detection of *in vivo* SUMO2/3-modification. SUMO2/3 β-catenin conjugates are identified with asterisks. (**D**) Whole cell lysates from treated MCF10-2A cells were run on SDS-PAGE and immunoblotted for the expression of the indicated proteins; β-actin served as loading control.

Nuclear exclusion of Axin1 would potentiate nuclear accumulation of β-catenin as it is no longer able to direct it to the destruction complex. Reduction of SENP7S increases recruitment of β-catenin to the nucleus (Fig. 5A,B and Supplementary Fig. S6). This β-catenin nuclear translocation is observed in multiple MCF10-2A cells following SENP7S knockdown and is significantly higher than in cells treated with non-targeting siRNA (*p* < *0.01*, Fig. 5B). Specifically, SUMOylated β-catenin is chromatin bound with siRNA-targeted knockdown of SENP7S (Fig. 5C). Consistently, the same conditions increase the active β-catenin (Fig. 5D). As chromatin-bound β-catenin initiates transcription of multiple cell cycle regulators and/or oncogenes, the expression of several β-catenin-regulated genes was evaluated. Consistently SENP7S loss induces c-Myc, Cyclin D1, and Aurora Kinase A levels (Fig. 5D). Induction of this gene set is associated with greater BCa recurrence and probability of distant metastasis in patients with early stage BCa (Stage I, lymph node negative, Supplementary Fig. S7).

**Figure 6:**
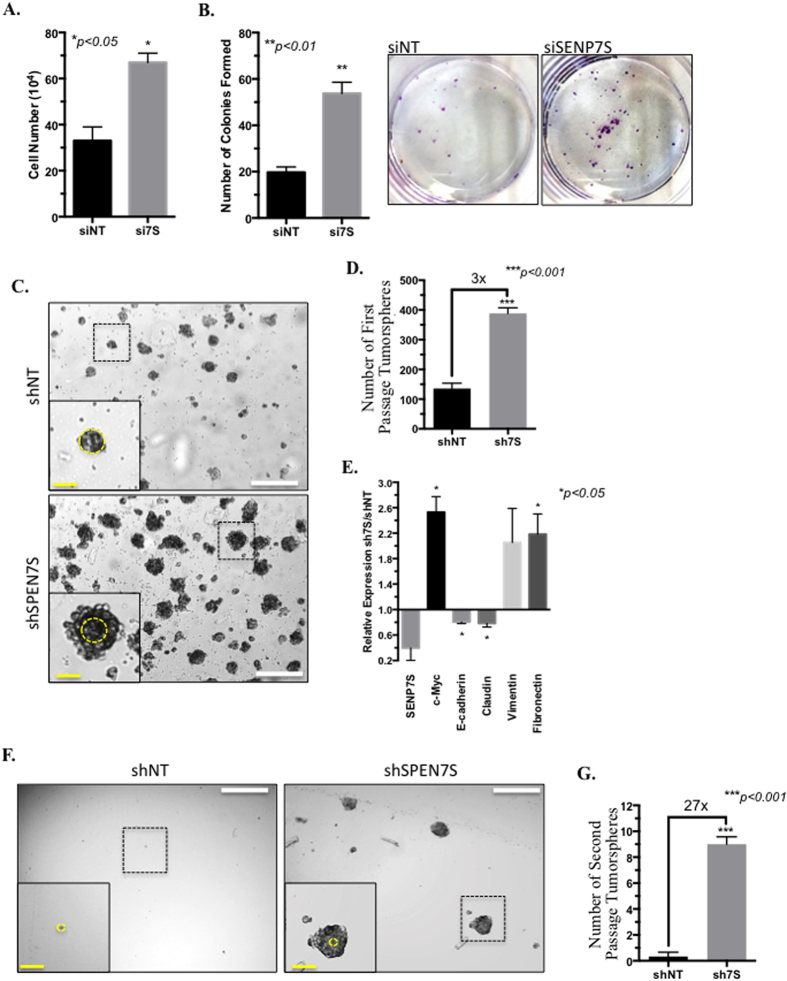
HyperSUMO conditions initiate tumorsphere formation. (**A**) After 72 hr siRNA treatment, MCF10-2A cells were evaluated for viability using a cell counter. All experiments were performed in duplicates. The graph represents the mean ± SEM of 3 independent experiments. (**B**) To test for propagation/colony formation abilities of normal MCF10-2A cells in the presence and absence of SENP7S, 1000 single cells were grown for 5 days after siNT or siSENP7S treatment and colonies (visible in the accompanying photograph) were counted. Student’s t-test indicates that knockdown of the large SENP7S population in MCF10-2A cells significantly enhances colony formation. (**C**) Single cell suspension of the appropriate stable SENP7S-deficient (shSENP7S) and non-targeting shRNA (shNT) MCF10-2A cells was generated. Ten thousand cells were grown in nonadherent media conditions for 8 days and imaged. White and yellow bars represent 500 μm and 100 μm, respectively. Tumorspheres highlighted with a black-dashed box were magnified in the insert. Yellow circle illustrates the size difference between shNT versus shSENP7S spheroids. (**D**) Total number of tumorspheres was counted in 3 random magnification fields for 3 independent experiments. Graph illustrates mean ± SEM and statistical significance using Student’s t-test. (**E**) First passage tumorspheres were harvested, RNA purified, and subjected to real-time PCR for assessment of the indicated mRNA. The fold-change in the indicated mRNA (2^ΔΔCt^ values) is shown; raw ΔCt values were used for Student’s t-test analysis of shSENP7S versus shNT tumorspheres. (**F**,**G**) Single cell suspensions of first passage tumorspheres were generated and cultured in nonadherent conditions to test for formation of second-generation spheroids. After 10 days, images were taken and the number of tumorspheres was assessed as described above (**D**).

Downregulation of SENP7S significantly increases cell counts (*p* < *0.05*, Fig. 6A) and the double time of MCF10-2A cells (Supplementary Fig. S8). Also increased anchorage-dependent colony formation is readily observed with multiple siRNA-targeting SENP7S in MCF10-2A (Fig. 6B).

Stable SENP7S-deficient MCF10-2A cells were generated and tumorsphere formation assays were utilized to assess anchorage-independent growth, a hallmark of cell transformation. Normal MCF10-2A and MCF10-2A expressing non-targeting shRNA do not grow efficiently in the non-adherent spheroid conditions. However, elevated SUMO-PTM with SENP7S-shRNA treatment initiates mammary epithelial cell transformation, larger spheroid size, and greater number of tumorspheres (Fig. 6C,D) suggesting that hyperSUMO conditions support tumor-initiating cell (TIC) proliferation. Additionally, passaging of the tumorspheres demonstrates that the SENP7S loss increases the self-renewal properties of the MCF10-2A cells (Fig. 6C,D). Transcriptome analysis of the spheroids revealed an induction of c-Myc and mesenchymal genes vimentin and fibronectin in SENP7S-deficient MCF10-2A cells (Fig. 6E). In contrast, epithelial genes E-cadherin and claudin are lost in these same tumor spheroids (Fig. 6E). Hence, the loss of SENP7S in MCF10-2A spheres initiate epithelial-mesenchymal transition and a more aggressive phenotype. Consistently, the SENP7S-deficient MCF10-2A spheroid can regenerate from a single cell population or be passaged (Fig. 6F,G); hence the loss of SENP7S potentiates the self-renewal properties of noncancerous mammary epithelial cells.

## Discussion

Assessment of NCBI’s RefSeq databases indicates multiple transcript variants for 4 of the 6 mammalian SENP, specifically SENP1, SENP5, SENP6, and SENP7. One SENP1, 1-SENP5, 1-SENP6, and 4-SENP7 isoforms include a catalytic domain analogous to the full-length transcript; hence these variants are likely active SUMO isopeptidases. Alternative splicing of the *SENP* transcripts occurs predominantly at exons close to the translational start site and consequently generates isoforms with distinct N-terminus regions. The N-terminus of SENP defines subcellular localization and enzyme substrate specificity. Therefore, these shorter isoforms may exhibit a different substrate profile and biological function compared to their respective full-length proteins. However, the functions of these SENP isoforms remain undefined. Our current report demonstrates the SENP7S isoform has a distinct expression, subcellular localization, and substrate profile compared to full-length SENP7L.

SENP7 is highly transcribed in NME with SENP7S as the predominant SENP7 isoform (Fig. 1B). SENP7S mRNA is significantly reduced early in breast carcinogenesis with SENP7S transcript decreased approximately 50% in DCIS (Fig. 1C). Similarly, SENP7S mRNA and protein is lost in samples from patients with multiple BCa subtypes and invasive carcinoma (Fig. 1D,E). Concurrently, in invasive carcinoma, SENP7L protein is elevated (Supplementary Fig. S2C,D). These changes in protein expression are consistent with our previous results that demonstrate reduced SENP7S mRNA and concurrent elevated SENP7L mRNA in samples from patients with metastatic BCa^4^. A global study on alternative splice transcripts in human breast tissue showed induction of SENP7 transcripts and exon-6 inclusion in multiple BCa subtypes^18^. Collectively, the data supports an inverse relationship between SENP7S and SENP7L.

We previously established that the PxVxL sequence in exon-6 facilitates interaction with the chromatin binding protein CBX5/HP1α^4^. Garvin *et al*. demonstrated this motif is required for nuclear localization of the full-length SENP7 as site-directed mutagenesis of this domain initiates cytosolic distribution of SENP7 in HeLa cells^15^. Exon-6 and consistently HP1α-interaction is missing for the SENP7S variant^4^. However, SENP7S does include a nuclear export sequence (amino acids 149–158) that prompts translocation to the cytosol. Only two additional deSUMOylases translocate to the cytosol; specifically SENP1 and SENP2 shuttle between nuclear and cytosolic subcellular compartments^19^,^20^. Interestingly, cytosolic SENP2 is readily ubiquitylated and targeted for proteasome-mediated degradation^20^. Therefore, it is unlikely to impact the dynamics of the multiple SUMOylated substrates in the cytosol. In contrast, SENP7S is localized less in the nucleus but predominantly in the cytosol of noncancerous and cancerous mammary epithelial cells (Fig. 2A and Supplementary Fig. S2).

**Figure 7:**
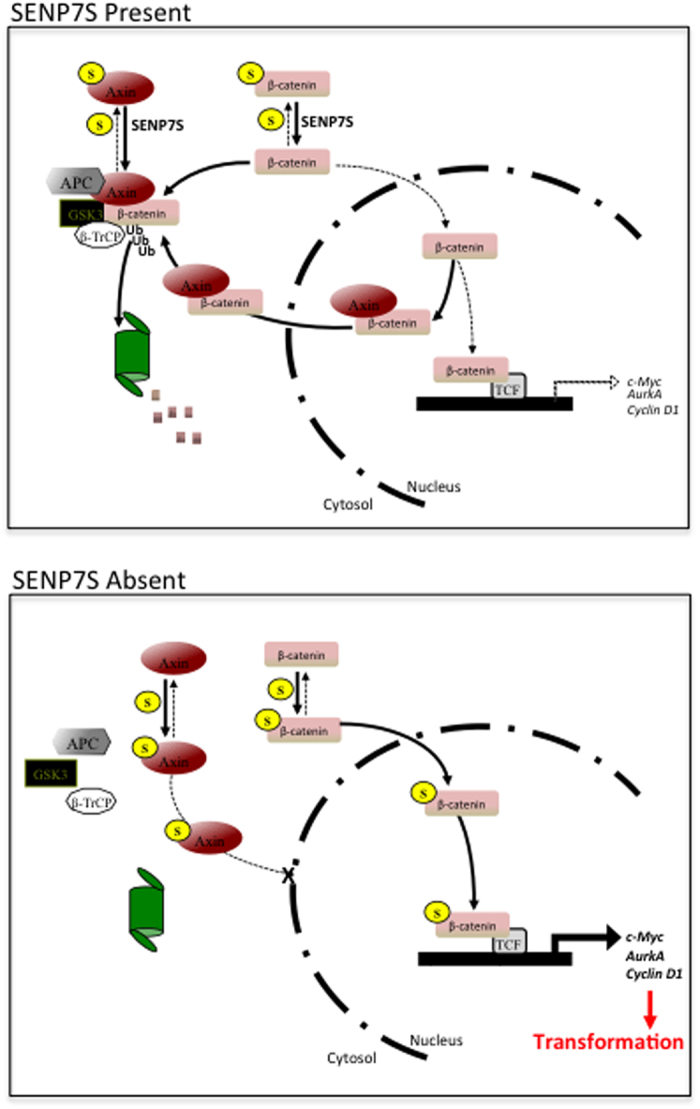
Schematic illustrating altered β-catenin signaling in the presence/absence of SENP7S in normal mammary epithelia. A detailed description is provided in the text.

Like SENP7L, SENP7S processes SUMO2/3 conjugates as targeted SENP7S knockdown potentiates SUMO2/3 conjugates. SUMOylated protein targets of SENP7L include HP1α, SUMO E3 ligase PC2, and transcription factor KAP1^4^,^15^,^16^,^17^. In contrast, SENP7S is inefficient at deSUMOylating HP1α even when overexpressed^4^. Collectively, our data shows SENP7S dictates SUMOylation and SUMO-dependent changes of β-catenin and Axin1. First, reduction of endogenous SENP7S potentiates SUMO2/3 chain formation on β-catenin and disrupts interaction with Axin1 (Figs 3A,C and 5C). Overexpression of SENP7S efficiently restores the lost β-catenin-Axin1 binding in SENP7S knockdown cells. Second, in the absence of SENP7S, Axin1 is subject to SUMO-PTM and this modified Axin1 is sequestered in the cytosol (illustrated in Fig. 7). As translocation of Axin1 to the nucleus is critical for export of nuclear β-catenin^21^, β-catenin accumulates in the nuclear compartment.

Translocation of Axin1 requires 1 nuclear export and 3 nuclear localization signals (NES and NLS, respectively^21^,^22^) with the strongest bipartite NLS sequence at amino acids 407–418 of the human Axin1^21^. Interestingly, sequence analysis using multiple SUMO predicting databases (GPS-SUMO and SUMOplot) indicated the presence of a non-consensus SUMOylation site from 407–410 a.a. It is highly probable that SUMO modification perturbs importin-recognition of the NLS and initiates subsequent cytosolic Axin1 accumulation. Additional experiments beyond the scope of this manuscript are required to evaluate changes in structure of SUMOylated Axin1.

As illustrated in Fig. 7, SENP7S modulates SUMO-PTM of Axin1 and β-catenin; preventing excess SUMOylation of either substrate in normal mammary epithelial cells. Consistently, β-catenin is readily targeted to the destruction complex, ubiquitylated, and degraded. Hence, excessive β-catenin levels and active β-catenin signaling is attenuated.

In the absence of SENP7S, Axin1 and β-catenin are hyper-SUMOylated (Fig. 7). SENP7S downregulation elicits induction of transcriptionally active nuclear β-catenin and prompts a response analogous to activation of Wnt signaling. Nuclear accumulation of β-catenin regulates transcription of multiple target genes; specifically it activates transcription of oncogenes c-Myc^6^,^23^, cyclin D1^24^, and Aurora kinase A^25^. Consistently, SENP7S loss enhances expression of these oncogenes (Fig. 5D). Simultaneous induction of these 3 genes is associated with onset of aggressive BCa, specifically a greater probability of disease recurrence and distant secondary tumor formation (Supplementary Fig. S7).

Noncancerous MCF10-2A cells do not propagate in spheroid conditions and consistently shNT MCF10-2A cannot be passaged (Fig. 6F). However, β-catenin-induced epithelial-mesenchymal transition is observed in SENP7S deficient spheroids as altered expression of vimentin^26^, fibronectin^27^, E-cadherin^28^, and claudin transcripts occurs (Fig. 6E). Stable SENP7S knockdown potentiates anchorage-independent growth and self-renewal properties (Fig. 6D,G, respectively) consistent with cell transformation.

## Methods

### Cell Culture and Stable Cell Generation

MCF10-2A and MCF7 were purchased from ATCC (Manassas, VA, USA). MCF10-2A cells were grown in DMEM:F12 media including supplements EGFR, cholera toxin, insulin, hydrocortisone, 5% horse serum, and 0.5% penicillin/streptomycin. MCF7 cells were cultured in DMEM media with 10% FBS and 1% penicillin/streptomycin.

The SENP7S-siRNA oligo was integrated into a puromycin-selection shRNA vector (Origene) to generate SENP7S-targeting shRNA (shSENP7S). Subsequently, stable shSENP7S MCF10-2A clones were selected based puromycin resistance; a similar approach was utilized with a control non-targeting shRNA vector (shNT).

### Transfections and siRNA treatments

Transient transfection in MCF10-2A cells of empty pcDNA vector and SENP7S plasmid was performed with Lipofectamine 2000 according to manufacturers’ instructions (Invitrogen). For siRNA studies, cells were transfected with commercially available non-targeting siRNA (siNT) and custom SENP7S siRNA (siSENP7S: AAGCCTTAATTTATCTGAAAGGG) using Dharmafect-2 reagent as directed (GE Life Sciences).

### SENP7S and SENP7L Antibodies

SENP7S and SENP7L antibodies were generated with assistance from Epitomics (Burlingame, CA). Specifically four rabbits were immunized with a peptide fragment for SENP7S (red bar, Fig. 1A) or SENP7L (yellow bar, Fig. 1A), respectively. Antibodies were then tested via Western Blot analysis of MCF7 treated with and without SENP7 siRNA as previously described^4^ (Supplementary Fig. S2A). Additionally, immunofluorescence studies were conducted on MCF7 cells transiently transfected with either Flag-tagged SENP7S (Fig. 2A) or SENP7L (Fig. 2B). Cells were incubated with either anti-Flag antibody or an isoform specific antibody.

### Real-time PCR

TissueScan Breast Cancer Survey II (Origene) was used for assessment of mRNA in human patient samples. For tumorsphere studies, total RNA was extracted from the first passage tumorspheres for both shSENP7S and shNT MCF10-2A cells using PureLink RNA Mini Kit (ThermoFisher Scientific) and converted to cDNA with iScript™ cDNA Synthesis Kit (BioRad) according to the manufacturer’s instructions. Real- time PCR was conducted using the iTaq™ Universal SYBR^®^ Green Supermix (BioRad) and 7500 Fast Real-Time PCR System (Applied Biosystems) with the indicated primers (Supplementary Table S1). Specifically, the first primer for SENP7S is designed to span Exon 5 (19-bases) and Exon 7 (8-bases) and the second primer identifies a sequence on Exon 7 (21 bases). SENP7L primers target the Exon 5 and Exon 6 while Taqman SENP7 primers target Exon 20-21.

Data is normalized to the internal control (actin). Samples were run in triplicates and either 2^−ΔCT^ or 2^−ΔΔCT^ values for the relative mRNA expression were calculated.

### Immunohistochemistry (IHC) for Human Tissue

IHC with SENP7L and SENP7S antibodies was performed on normal versus invasive ductal carcinoma 2-mm tissue samples purchased from Pantomics (n = 18 and 19, respectively). The IHC and hematoxylin-counterstain procedures were conducted as previously established^29^. SENP7-specific staining was validated against mock IgG control. Total cell nuclei were counted in random 40X magnification fields; SENP7 isoform positive cells were distinguished from negative cells.

### Subcellular Fractionation

Transfected cells were harvested and subject to subcellular fraction according to manufacturers instructions for Protein Fractionation kit (Thermo Fisher scientific). Isolated fractions were resolved via SDS-PAGE and Western blot analysis.

### Immunoprecipitation and Chromatin-Immunoprecipitation (CB/IP)

Isolated MCF10-2A cells were subject to immunoprecipitation and CB/IP as presented in detail in a recent publication^30^.

### SDS-PAGE and Western Blots

Proteins in the whole cell lysates and immunoprecipitated samples are denatured with a loading buffer that includes SDS. Then SDS-PAGE and Western blot analysis was performed as described previously^4^. Uncropped blots for Western blot images included in the main-text are provided in Supplementary Fig. S9.

### Immunofluorescence (IF)

MCF10-2A cells were seeded on coverslips and transfected as indicated. After 24 hr, cells were fixed in 4% paraformaldehyde at 4 °C, permeabilized with 0.02% Triton X-100 for 10 mins, and blocked in a 2% BSA/goat serum solution. Samples were incubated with anti-Axin1 or β-catenin monoclonal antibodies (Cell Signaling) and subsequently stained with DAPI. The images were acquired using the Olympus BX51 microscope and subsequently analyzed by Image J.

### Cell Proliferation and Clonogenic Assay

MCF10-2A cell proliferation was evaluated as previously described^16^. For the clonogenic assay to test anchorage-dependent proliferation, siRNA-treated MCF10-2A cells were first dissociated to generate single cells. Then, 500 cells were seeded in a 6-well plate and after 10-days, the cell colonies were fixed and stained with crystal violet. For each experiment, the total number of colonies was counted.

### Tumor Spheroid Studies

Single cell suspension was generated with 10,000 cells; subsequently single cells were plated on PolyHEMA (Poly 2-hydroxyethyl methacrylate) coated plates. Cells were grown in non-adherent conditions with MammoCult media (Stemcell) supplemented with MammoCult proliferation supplement (Stemcell), Heparin (Sigma-Aldrich), Hydrocortisone (Sigma-Aldrich), Methylcellulose (Sigma-Aldrich), and Penicillin Streptomycin (ThermoFisher scientific) for 10 day. To test self-renewal, mammospheres were passaged; specifically, spheroids were dissociated to generate a single cell suspension and 20,000 cells were reseeded.

### Statistical Analysis

Results are expressed as mean±SEM. All data was analyzed with GraphPad Prism. Student’s t-test or ANOVA followed by Tukey’s posthoc test was used to calculate *p* values; *p-*values < 0.05 was considered statistically significant.

## Additional Information

**How to cite this article:** Karami, S. *et al*. Novel SUMO-Protease SENP7S Regulates β-catenin Signaling and Mammary Epithelial Cell Transformation. *Sci. Rep.* **7**, 46477; doi: 10.1038/srep46477 (2017).

**Publisher's note:** Springer Nature remains neutral with regard to jurisdictional claims in published maps and institutional affiliations.

## Supplementary Material

Supplementary Information
