# Supplementary material for: Novel SUMO-Protease SENP7S Regulates β-catenin Signaling and Mammary Epithelial Cell Transformation
Source: Sci Rep. 2017 Apr 21;7:46477. doi: 10.1038/srep46477 (PMC5399363; doi:10.1038/srep46477)
Supplement: Supplementary Information [file srep46477-s1.pdf]

Novel SUMO-Protease SENP7S Regulates β-catenin Signaling and Mammary Epithelial Cell Transformation

Samaneh Karami[1], Feng-Ming Lin[2,3], Santosh Kumar[1], Shaymaa Bahnassy[1], Hariprasad Thangavel[1], Maram Quttina[1], Yue Li[4], Jing Ren[1], and Tasneem Bawa-Khalfe[1]*

Supplementary Information:

Transcript	Primer
SENP7S	5’-GCCTTAATTTATCTGAAAGGGGCTCAC-3’<br>5’-GGTCTTCAGGCTGAGTATCAG-3’
SENP7L	5'- CAATCAGACTCATTGCCTTCG -3‘<br>5'- GTTCCCAGGACATTCGTCAAT A -3’
SENP7	TaqMan Gene Expression Assay (ABI)<br>Hs00221046_m1
c-Myc	5'-CTGGTGCTCCATGAGGAGAC-3’<br>5'-CTTTTCCACAGAAACAACATC-3’
E-cadherin	5‘- GAGAGGAATCCAAAGCCTCAGGT-3’<br>5‘-CTGGTTATCCATGAGCTTGAGAT-3’
Claudin	5’-GGCCGGCCTTATGGTGATA-3’<br>5’-GCCACCAGCGGATTGTAGA-3’
Vimentin	5’-TACAGGAAGCTGCTGGAAGGCG-3’<br>5’-TGGCAGAGGCAGAGAAATCCTGC-3’
Fibronectin	5’-CCGCCGAATGTAGGACAAGA-3’<br>5’-TGCCAACAGGATGACATGAAA-3’

Supplementary Table S1: Primers used for the detection of the indicated transcript.

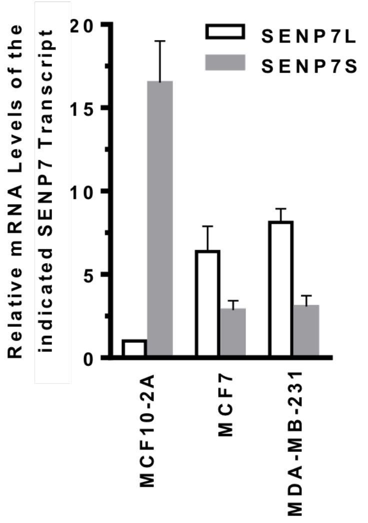

Supplementary Figure S1: SENP7 isoforms in cultured noncancerous and BCa cell lines. Normal mammary epithelial cells, MCF10-2A exhibit greater SENP7S than SENP7L while the inverse is true for the MCF7 and highly metastatic BCa cell lines MDA-MB-231 (n=2-3). Data in represents the fold-change of the indicated transcript in cancer cells as compared to MCF710-2A cells.

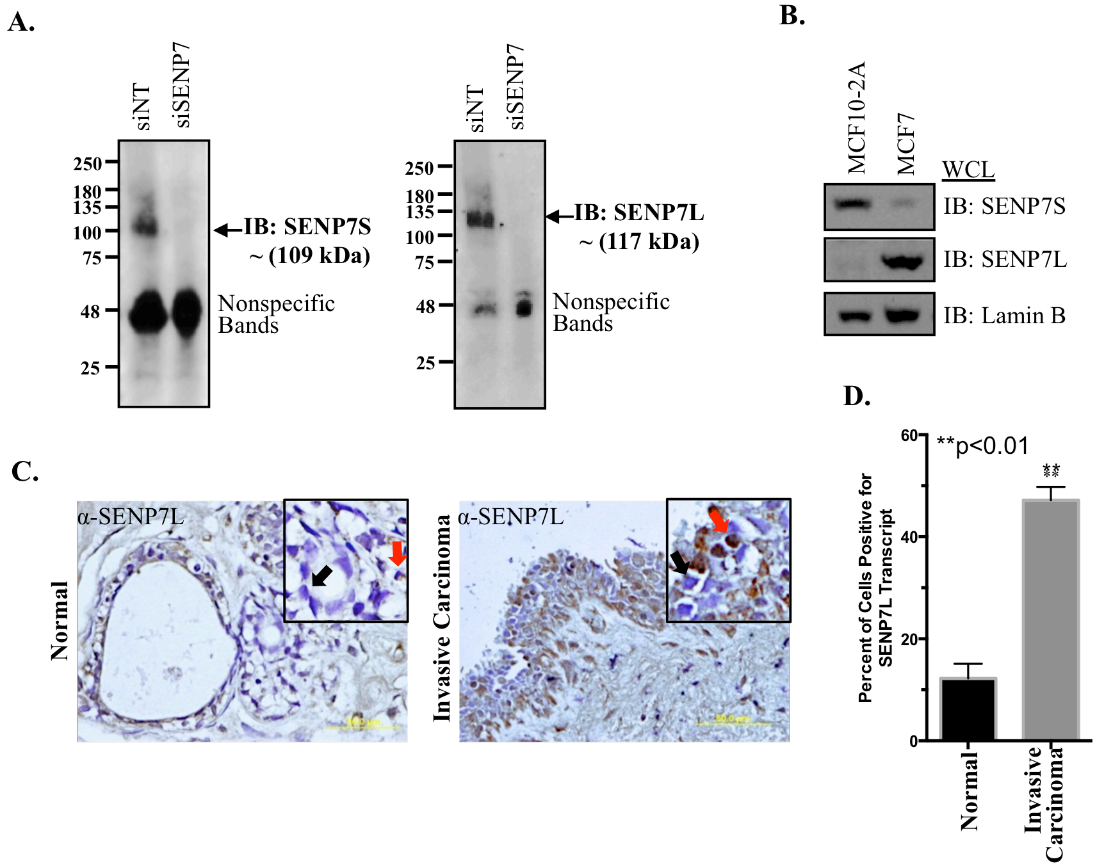

Supplementary Figure S2: Expression of SENP7 isoforms in BCa. (**A**) MCF7 cells were subject to 48hr treatment with either non-targeting or SENP7-targeting siRNA and whole cell lysates were run on SDS-PAGE. SENP7S or SENP7L antibodies identified the respective SENP7 isoform at the predicted molecular weights indicated in siNT, but not siSENP7 samples. (**B**) Whole cell lysates from MCF10-2A and MCF7 were evaluated for SENP7S vs. SENP7L protein levels using specific antibodies with Lamin B serving as a control. (**C**) IHC with SENP7L antibody was used to evaluate SENP7L levels in human tissue samples (normal, n=18 and carcinoma, n=19). Red arrows indicate SENP7L-positive cells and black arrows highlight SENP7L-negative cells. (**D**) The mean±SEM of the percent of SENP7L-positive to total cells in normal (n=5) and carcinoma (n=7) is presented in the graph.

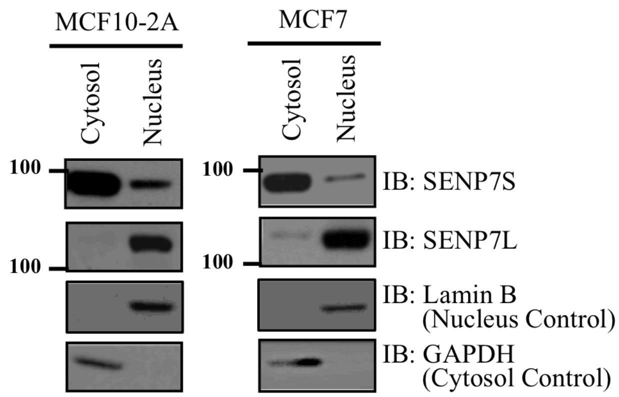

Supplementary Figure S3: Subcellular distribution of SENP7 isoforms in noncancerous and BCa cells. Cells from both established mammary epithelial lines MCF10-2A and MCF7 were harvested to isolate cytosolic and nuclear fractions.

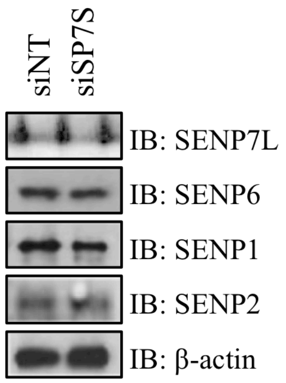

Supplementary Figure S4: SENP7S siRNA does not affect expression of other nucleoplasm SENP. Whole cell lysates from MCF10-2A cells treated with non-targeting or SENP7S-targeting siRNA were harvested and subject to Western blot analysis. Immunoblots for and SENPs located in the nucleoplasm are shown.

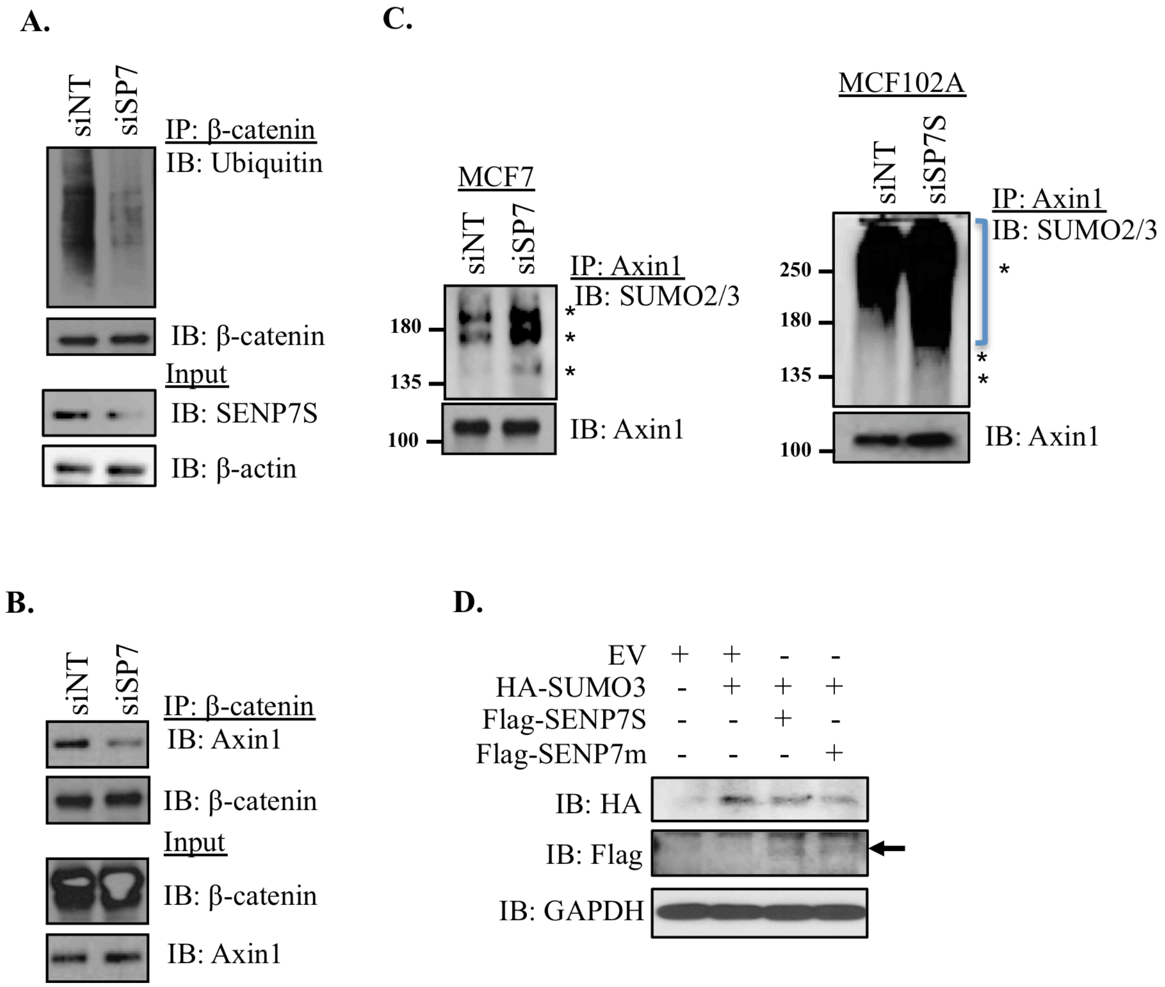

Supplementary Figure S5: SENP7 knockdown reduces β-catenin ubiquitylation and Axin1-binding while increasing Axin SUMOylation. MCF7 cells were subject to 48hr treatment with either non-targeting or SENP7-targeting siRNA. (**A**). Whole cell lysates were run on SDS-PAGE and immunoblots for SENP7S confirmed knockdown efficiency. Additionally, cell lysates were incubated with β-catenin antibody and immunoprecipitated β-catenin was evaluated for ubiquitin-PTM. (**B**) β-catenin immunoprecipitates were evaluated for association with Axin1. All immunoblots are representative of two independent experiments. (**C**) MCF7 and MCF102A cells were treated with non-isoform specific SENP7 (siSP7) and SENP7S-specific siRNA, respectively. After 48 hour,

endogenous SUMO2/3 modification of Axin1 was observed in both cell lines highlighted with asterisks. (**D**) Expression of the indicated plasmids used for **Fig. 4C** is demonstrated via SDS-PAGE and immunoblots with antibodies for either the HA or Flag-tag.

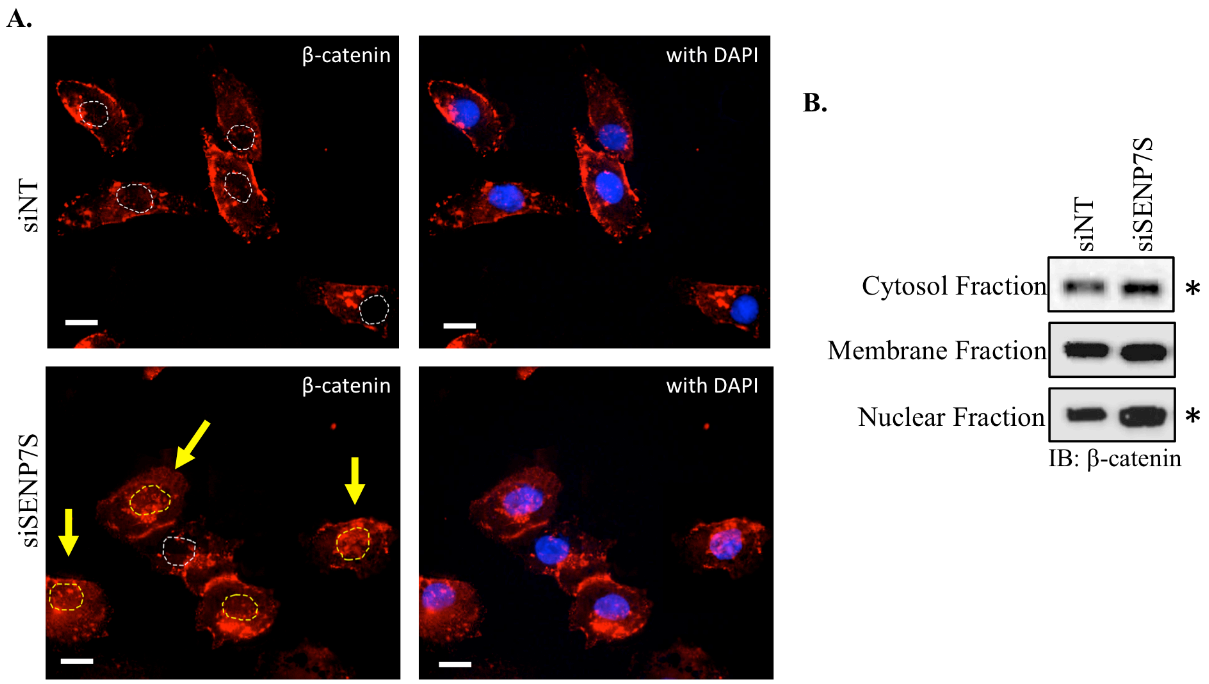

Supplementary Figure S6: Nuclear β-catenin translocation following knockdown of SENP7S in multiple MCF10-2A cells. (**A**) Immunofluorescence studies were conducted to evaluate β-catenin localization. After immunostaining, 3-4 images were acquired at 60X magnification of β-catenin (red) with and without overlay of DAPI (blue). An outline of the DAPI-positive region was drawn to highlight the nucleus (dashed line) and β-catenin compartmentalization was assessed. Image shows the population of cells with β-catenin inside (yellow dashed lines and yellow arrows) versus outside (white dashed lines) the nucleus; the white bar indicates 10μM. (**B**) Fractionated samples from **Fig. 4B** were immunoblotted for β-catenin. Asterisks highlights accumulation of β-catenin in the indicated cell fractions.

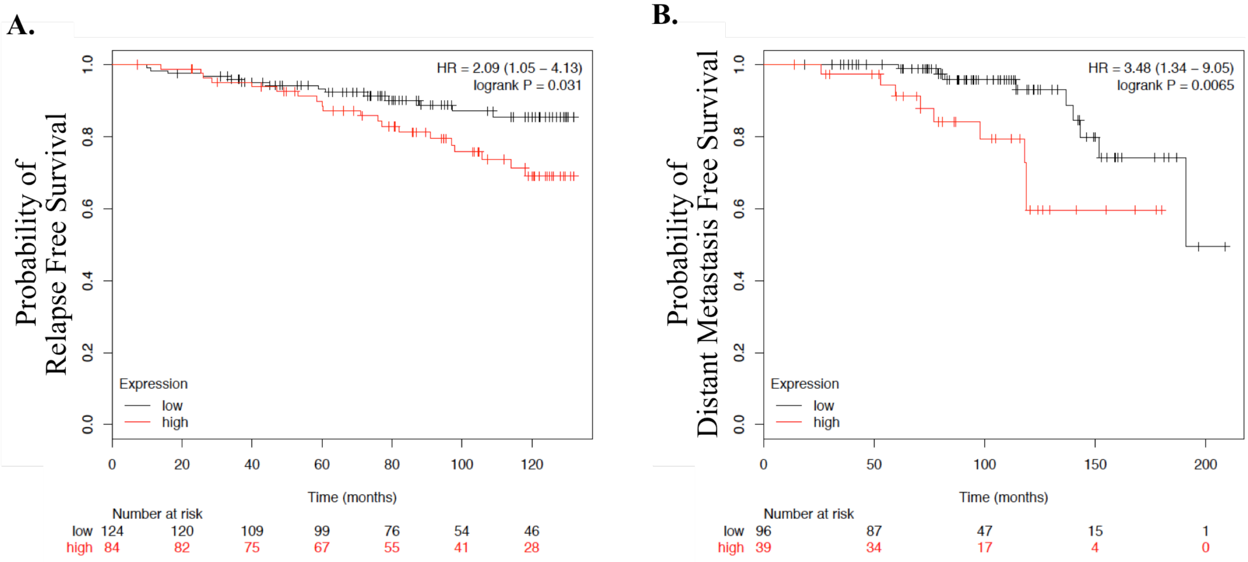

Supplementary Figure S7: Correlation of cMyc, Cyclin D1, and Aurora kinase A and onset of aggressive BCa. Kaplan-Meier curves were generated to evaluate how high versus low expression of the gene set (cMyc, Cyclin D1, and Aurora kinase A) impacts the probability of relapse-free survival (n=208, **A**) and distant metastasis free survival (n=135, **B**). Publically available samples of patients with the early stage cancer (Stage 1) with no lymph node metastasis were selected and analyzed via kmplot.com.

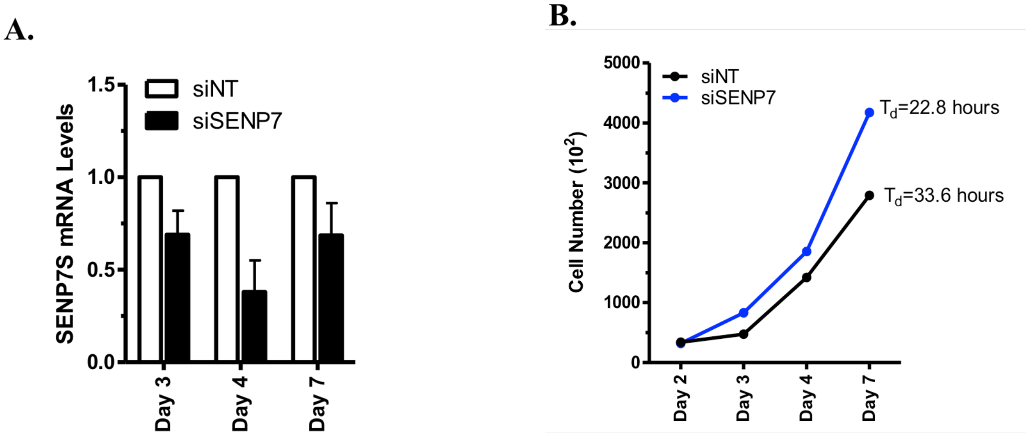

Supplementary Figure S8: SENP7S knockdown affects MCF10-2A cell growth. (**A**) The mRNA levels of SENP7S after targeted and non-targeted siRNA treatment (siSENP7 and siNT, respectively) in subsequent MCF10-2A cell studies. (**B**) Equal amounts of MCF10-2A cells treated with SENP7 siRNA (siSENP7) or non-targeted siRNA (NT-siRNA) were harvested at the indicated time points. The number of cells is presented in the graph; the calculated doubling rate (Td) is higher following SENP7 knockdown versus NT-siRNA control treatment.

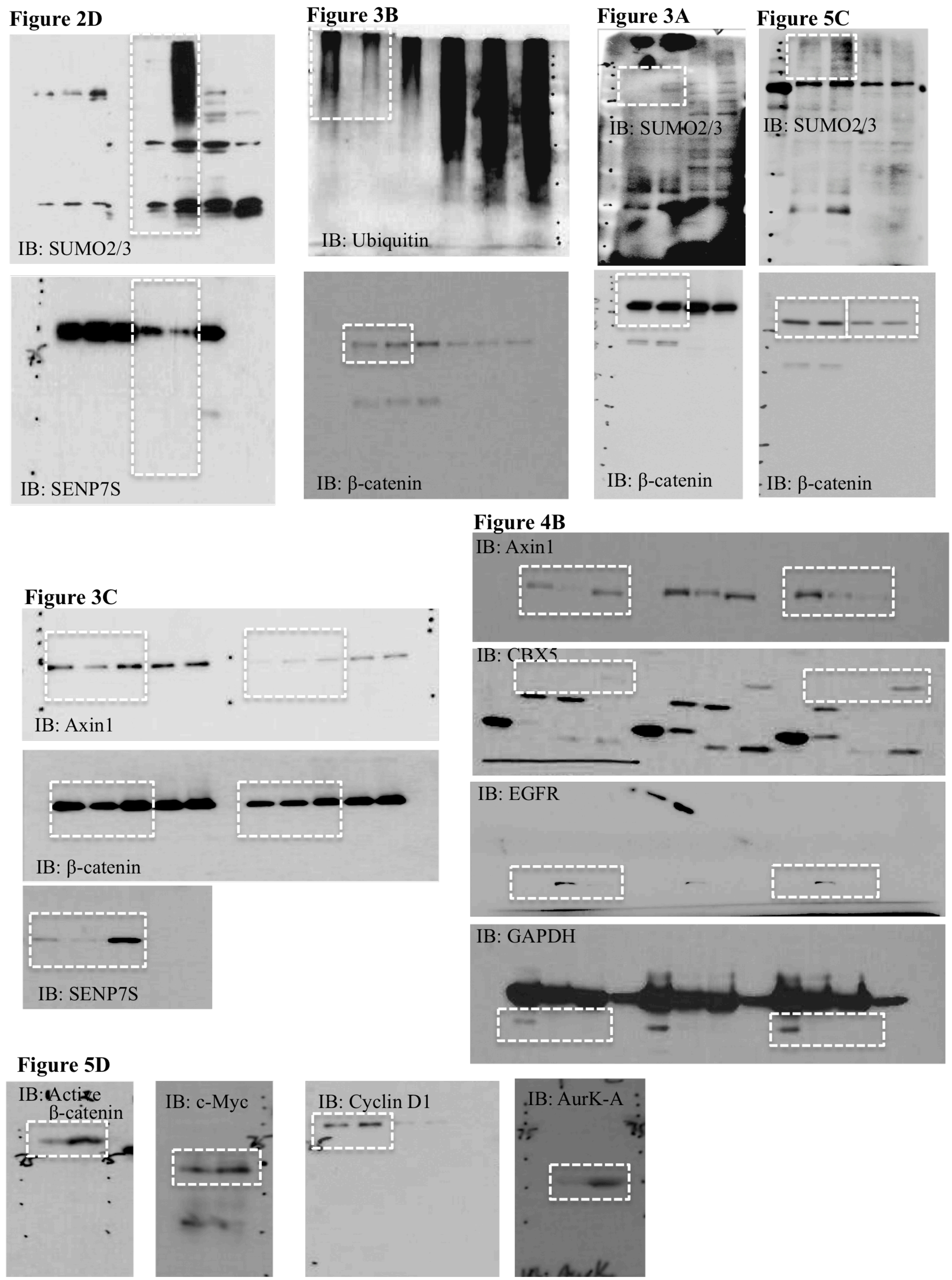

Supplementary Figure S9: Uncropped Blots for images presented in the main text.
